# Medical Appointment

**Published:** 1851-10

**Authors:** 


*Medical Appointment.*—Paul F. Eve, M. D., for many years Professor of Surgery in the Georgia Medical College, at Augusta, and late of the medical department of the University of Louisville, Ky., has recently been appointed to the Chair of Surgical Anatomy and Clinical Surgery in the medical department of the University of Nashville, Tenn. Professor Eve is an experienced and able teacher, as well as an eminently skillful Surgeon. The Medical School at Nashville has been fortunate in securing his services.

